# A systematic review and meta-analysis of psychological predictors of successful assisted reproductive technologies

**DOI:** 10.1186/s13104-017-3049-z

**Published:** 2017-12-07

**Authors:** S. Purewal, S. C. E. Chapman, O. B. A. van den Akker

**Affiliations:** 1 Institute of Psychology, Faculty of Education, Health and Wellbeing, University of Wolverhampton, Wolverhampton, WV1 1AD UK; 2 Department of Pharmacy & Pharmacology, University of Bath, Claverton Down Road, Bath, BA2 7AY UK; 3 Department of Psychology, School of Science and Technology, Middlesex University, London, NW4 4BT UK

**Keywords:** Infertility, Assisted reproductive technologies, Psychology, Depression, Anxiety, Meta-analysis

## Abstract

**Objectives:**

The aim of this systematic review and meta-analysis was to perform an updated investigation of the effects of depression and anxiety on pregnancy outcomes following assisted reproductive technologies. A bibliographic search was performed using PubMed, PsycINFO, Embase, Science Direct databases. Data retrieved were analysed using a random effects model to estimate standardised mean differences.

**Results:**

Of the 22 included studies, 18 investigated depression, 15 state anxiety, and seven trait anxiety. Data from 4018 patients were included in the meta-analysis. Results indicated that women who achieved pregnancy or a live birth reported lower levels of depression pre-treatment than those who did not, although the effects were small d = − 0.177 (95% CI − 0.327 to − 0.027, z = 2.309, p = 0.021). These results were consistent under different methodological conditions and the quality of these observational were graded as satisfactory. A similar pattern was seen for state (d = − 0.096, 95% CI − 0.180 to − 0.012: z = 2.241, p = 0.025) and trait anxiety (d = −  0.188, 95% CI − 0.007 to 0.356, z = 2.181, p = 0.029). More research is needed to investigate the impact of psychological variables on assisted reproductive technologies outcomes and moderator influences during assisted reproductive technologies processes.

**Electronic supplementary material:**

The online version of this article (10.1186/s13104-017-3049-z) contains supplementary material, which is available to authorized users.

## Introduction

According to The Human Fertilisation and Embryology Authority (HFEA), in 2014 [1], 2% of all the babies born in the UK had been conceived through In vitro fertilisation (IVF) treatment. Studies since the 1980s [2, 3] have been reporting that some patients find IVF stressful and psychological burden are common reasons why some couples stop treatment [4]. However, previous synthesis of this research has found no or small impact of psychological variables on assisted reproductive technologies (ART) outcomes. Boivin et al. [5] found that emotional distress was not related to ART outcomes. Whereas, Matthiesen et al. [6] found a small effect size for stress, state and trait anxiety and decreased clinical pregnancies.

Despite Boivin [5] and Matthiesen’s [6] reporting no or little impact of psychological distress in ART outcomes, meta-analysis/systematic reviews on the effectiveness of psychological interventions in reducing psychological distress and promoting pregnancy rates have reported conflicting data. One meta-analytic review [7] reported that psychological interventions were not effective at reducing depression or anxiety but they did improve pregnancy rates. Whereas, a recent critical review [8] found psychosocial interventions improved psychological and pregnancy outcomes. However, Akioyamen et al. [9] found the use of antidepressants had no impact on fertility treatment pregnancy rates.

The aims of this meta-analysis were to perform an updated meta-analysis investigating the effects of depression, state and trait anxiety on ART outcomes.

## Main text

### Method

This paper presents part 1 of a two-part review that investigated lifestyle and Body mass index (BMI) predictors of ART outcomes (in press). The systematic review and meta-analysis was performed following PRISMA and MOOSE guidelines [10].

#### Eligibility criteria

Studies were considered if they presented original data and reported live birth rates or pregnancy outcome data. Studies were excluded if they did not investigate baseline (before stimulation) maternal depression, state anxiety (transitory state) or/and trait anxiety (stable disposition of anxiety-proneness) and ART outcomes. They were included if they used a standardised psychological measure (e.g., BDI—Beck’s Depression Inventory) reporting continuous or categorical (cut-off-score) data. Studies that assessed anxiety referencing the current time or a recent period (such as the last 2 weeks) were classed as ‘state’ anxiety e.g., the Psychological general well-being index (PGWB); Hospital Anxiety and Depression Scale (HADS) and the Zung Self-Rating Anxiety Scale because sensitivity data-analyses revealed no significant differences between the effect size of general anxiety scores to specific state anxiety scores (Q = 0.866, d.f. = 1, p = 0.352). We excluded studies assessing psychological variables after stimulation because ovarian stimulation leads to increases in stress hormones (i.e., serum norepinephrine and cortisol values) [11].

Only studies using ART techniques were included (e.g., IVF, intracytoplasmic sperm injection—ICSI, zygote intrafallopian transfer—ZIFT). Other exclusion criteria were if it was not possible to calculate unadjusted effect sizes for predictor variables (e.g., predictor data grouped by outcome, only adjusted data reported) and therefore meta-analysis of unadjusted effect sizes could not be achieved.

#### Information sources and search

We searched for relevant publications in six bibliographic databases—PubMed, PsycInfo, Embase, ScienceDirect, Web of Science and Scopus. In PubMed, the search used the following keywords and abstracts: (“Pregnancy”[Mesh] OR “Pregnancy” OR “pregnant” OR “live birth” OR “birth rate”) AND (“IVF” OR “intracytoplasmic” OR “intracytoplasmic sperm injection” OR “in vitro fertilization” OR “ICSI” OR “assisted reproductive technology” OR “in vitro fertilisation”) AND (“psychological stress” OR “depressive disorder” OR “anxiety” OR “anxiety disorder” OR “adjustment disorder” OR “emotions” OR “psychosomatic medicine” OR “psychological adaption” OR “distress” OR “depression” OR “stress” OR “occupation stress” OR “stressful life events” OR “major life events” OR “stressors”). We limited the searches to studies published after 1979/01/01 and conducted in humans. Hand searches of references cited in previous review papers were also conducted and the search was updated in November 2016.

#### Study selection, data collection process and data items

SP, OvdA and SC independently screened titles, abstracts and full-text reports [12]. Any disagreements were resolved by discussion. Data extracted included all independent (depression; state anxiety; trait anxiety) and dependent variables (live birth or pregnancy) and sample sizes. When two or more dependent variables were reported (e.g., serum pregnancy, clinical pregnancy and live birth), the data which was considered ‘gold standard’ and most relevant to patients was recorded (in this case, live birth) [13]. We also extracted patient, treatment and study characteristics.

#### Risk of bias

SP and OvdA independently assessed the quality of each study using Newcastle–Ottawa Scale (NOS) [14] and cross checked with each other to reach 100% consensus. The scale awarded a maximum of nine stars to each study: four stars for the adequate selection of cases and controls, two stars for comparability of cases and controls, and three stars for the adequate ascertainment of the exposure in both the case and control groups. We defined high quality as scoring at least seven stars the on the NOS; medium quality as scoring five or six stars and low quality as four or fewer stars.

#### Summary measures and synthesis of results

Comprehensive meta-analysis [15] was used to calculate overall weighted effect sizes using a random effects model. Extracted data (e.g., events, means) were converted into standardised mean differences and used to compare women with live birth/pregnancy outcomes and women without. Outliers were identified as studies with residuals greater than 1.96 and they were removed from the analysis as recommended.

#### Heterogeneity

We quantified heterogeneity in study effect sizes using the I^2^ statistic. We intended to conduct moderator analyses to investigate significant heterogeneity where more than 10 studies provided data on potential moderators (as recommended by guidelines; [16]). However, as shown below, insufficient studies were available, so moderator analyses were not performed.

#### Sensitivity analysis

Sensitivity analyses were conducted to examine whether effects were robust under different methodological assumptions: (1) when only live birth and only pregnancy data are included; (2) when only pregnancy ultrasound scan results and only pregnancy test results are used; (3) when only first time ART users data is included; (4) when results from a single cycle are used (not multiple cycles); (5) when only IVF, only ICSI and a combination of IVF and ICSI treatments are used: (6) when only high quality were included; and (7) when studies were recent (studies published from 2010 onwards were considered to be recent).

#### Publication bias

We tested for publication bias for by examining funnel plots for evidence of asymmetry, and using Duval and Tweedie’s trim and fill method to impute studies where evidence of asymmetry was present. We also tested for the significance of these effects using Egger’s *t* test.

### Results

#### Study selection

The screening process is summarised in the study PRISMA flow chart (Additional file 1: Figure S1). Where papers provided insufficient data for the meta-analysis, authors were contacted for additional data and three corresponding authors (D. Lancastle, K. Sanders and R. Türk) responded with additional, unpublished data.

#### Study characteristics

Of the 22 included studies, 18 studies investigated depression, 15 state anxiety, and seven trait anxiety. Data from 4018 patients were included in the meta-analysis. An overview of study characteristics is presented in Additional file 2: Table S1.

### Synthesis of results

#### Depression

Eighteen studies reported on depression [17–34], 2 were removed from these analyses as outliers [20, 28]. In the remaining studies there was a small, negative and significant effect of depression in women who achieved a pregnancy or live birth than in women who did not − 0.101 (95% CI − 0.193 to − 0.009, z = 2.152, p = 0.031). This estimate was not significantly heterogeneous (I^2^ = 24.956%, p = 0.176) see Additional file 3: Figure S2.

*Sensitivity analysis* The effects of depression remained consistent in the sensitivity analyses (see Table 1), with the exception that when the analysis was conducted in studies examining first time ART and in studies reporting IVF outcomes (not ICSI), effects became smaller and nonsignificant.

**Table 1:** Sensitivity analyses on depression data

	d [95% CI OR]	Heterogeneity (I^2^)
Pregnancy scan only (k = 11)	− 0.122 [− 0.212,− 0.031], z = 2.630, p = 0.009	12.401%, p = 0.326
First ART (k = 11)	− 0.077 [− 0.160, 0.006], z = 1.821, p = 0.069	< 0.001%, p = 0.568
Single cycle only (k = 10)	− 0.192 [− 0.391, − 0.011], z = 1.751, p = 0.080	28.499%, p = 0.182
Only IVF (k = 10)	− 0.045 [− 0.180, 0.089], z = 0.662, p = 0.508	18.879%, p = 0.269
ICSI and IVF (k = 5)	− 0.120 [− 0.245, 0.005], z = 1.887, p = 0.059	19.918%, p = 0.288
High quality (k = 9)	− 0.101 [− 0.200, − 0.002], z = 1.995, p = 0.046	11.922%, p = 0.335
Recent only (k = 5)	− 0.181 [− 0.324, − 0.037], z = 2.469, p = 0.014	< 0.001%, p = 0.472

All sensitivity data analyses are presented for the combined LB and pregnancy outcome except when separate pregnancy or livebirth outcomes are reported

#### State anxiety

Fifteen studies reported baseline state anxiety [17–19, 23–25, 27, 28, 30–33, 35–38]. Initial data analyses revealed one study was an outlier and their data was removed [28] from the analyses, there was a small, significant and negative effect of state anxiety between women who achieved live birth or pregnancy and women who did not − 0.096 (95% CI − 0.180 to − 0.012: z = − 2.241, p = 0.025) and no evidence of heterogeneity (I^2^ = 0.00%, p < 0.454) see Additional file 4: Figure S3.

*Sensitivity analysis* In subsequent state anxiety analyses, the evidence for state anxiety varies slightly under different methodological conditions. See Table 2 for all results.

**Table 2:** Sensitivity analyses on state anxiety data

	d [95% CI OR]	Heterogeneity (I^2^)
Pregnancy scan only (k = 9)	− 0.104 [− 0.199, − 0.008], z = 2.122, p = 0.034	< 0.001%, p = 0.647
First ART (k = 9)	− 0.086 [− 0.186, 0.013], z = 1.699, p = 0.089	< 0.001%, p = 0.775
Single cycle (k = 10)	− 0.061 [− 0.154, 0.032], z = 1.277, p = 0.202	< 0.001%, p = 0.488
IVF only (k = 8)	− 0.132 [− 0.318, 0.054], z = 1.393, p = 0.164	18.128%, p = 0.287
IVF and ICSI mixed (k = 5)	− 0.079 [− 0.187, 0.029], z = 1.439, p = 0.150	< 0.001%, p = 0.600
High quality (k = 6)	− 0.096 [− 0.207, 0.015], z = 1.693, p = 0.091	< 0.001%, p = 0.674)
Recent only (k = 6)	− 0.061 [− 0.170, 0.048], z = 1.097, p = 0.273	< 0.001%, p = 0.747

All sensitivity data analyses are presented for the combined LB and pregnancy outcome except when separate pregnancy or livebirth outcomes are reported

#### Trait anxiety

Across the seven studies reporting data on trait anxiety [27, 32, 33, 35–38] there was a significant difference between women who achieved a live birth or pregnancy and women who did not − 0.188 (95% CI − 0.007 to 0.356, z = − 2.181, p = 0.029) and no evidence of heterogeneity (I^2^ < 0.001%, p < 0.965) see Additional file 5: Figure S4.

*Sensitivity analysis* Analysis revealed that the evidence for trait anxiety was not robust under different methodological conditions. There were not enough studies to measure trait anxiety effect in high quality studies. See Table 3 for all results.

**Table 3:** Sensitivity analyses on trait anxiety data

	d [95% CI OR]	Heterogeneity (I^2^)
Pregnancy scan only (k = 3)	− 0.180 [− 0.404, 0.044], z = 1.577, p = 0.115	< 0.001%, p = 0.955
Pregnancy test only (k = 3)	− 0.267 [− 0.576, 0.043], z = 1.690, p = 0.091	< 0.001%, p = 0.883
First ART (k = 3)	− 0.172 [− 0.394, 0.051], z = 1.513, p = 0.130	< 0.001%, p = 0.989
Single cycle (k = 4)	− 0.153 [− 0.365, 0.058], z = 1.424, p = 0.155	< 0.001%, p = 0.965
IVF only (k = 5)	− 0.207 [− 0.446, 0.033], z = 1.693, p = 0.090	< 0.001%, p = 0.925
Recent studies only (k = 2)	− 0193 [− 0.455, 0.069], z = 1.446, p = 0.148	< 0.001%, p = 0.811

All sensitivity data analyses are presented for the combined LB and pregnancy outcome except when separate pregnancy or livebirth outcomes are reported

#### Publication bias

Data indicated low levels of publication bias risk. For the depression dataset, trim and fill data analyses revealed only 2 additional studies would be needed to ensure the funnel plot was generally symmetrical and Egger’s regression intercept was not significant t(14) = 0.352, p = 0.730. Trim and fill data analyses for state anxiety revealed no additional studies were needed, the funnel plot was symmetrical, but Egger’s meta regression intercept was also significant (− 1.08, 95% CI − 2.44, 0.281, p = 0.05). Trim and fill data analyses for trait anxiety revealed no additional studies were needed, the funnel plot was symmetrical and Egger’s meta regression intercept was not significant (− 0.169, 95% CI − 1.658, 1.319 p = 0.391).

### Discussion

Findings from this updated meta-analysis report that depression, state and trait anxiety have a small, significant and negative effect on ART outcomes, which were generally robust under different methodological assumptions. These results provide an updated review of the literature from Boivin et al. [5] and Matthiesen et al. [6], who reported little impact of psychological variables on ART outcomes. Cumulatively, these findings provide some encouragement to patients and clinicians, that baseline anxiety and depression will only have a small impact on their ART outcomes. However, for some patients this small impact could result in negative outcomes. Our results indicate that clinics could provide psychological support to minimise any psychological distress to help improve ART outcomes.

However, the research literature on the effects of depression and anxiety on ART outcomes is narrowly focused. That is, the studies included in this review often only measured whether depression and anxiety predict ART outcomes and did not acknowledge other factors that could impact the relationship between psychological variables and ART outcomes. For example, depressed patients are more likely to smoke or have a poor diet but the relationship is complex [39]. Smoking has consistently been found to be detrimental to ART outcomes [40–42] and the effect of obesity on ART outcome is inconsistent with some reviews reporting a negative impact [43–46], and other reviews finding only a small effect of obesity [47] or insufficient evidence to support an effect [48]. To the author’s knowledge, no review has examined whether psychological variables are moderated by lifestyle factors and BMI that could affect ART outcomes. The relationship between psychological variables and lifestyle factors such as smoking, alcohol and obesity is inter-related. Depression and anxiety are often comorbid with obesity and binge-eating [39, 49], and depression and anxiety are known to be comorbid with alcohol consumption [50] and smoking [51]. We confirm a substantial effect for BMI and smoking and ART outcomes independently [Authors, in press]. However, unlike BMI and smoking reviews, the numbers of studies and sample sizes included in psychological ART meta-analyses are much smaller.

To conclude, depression and anxiety have a small, significant and negative effect on ART outcomes. The clinical implications of this study are that some patients experiencing depression or anxiety may need psychological support before they start treatment, to help improve ART outcomes.

## Limitations

There is a gap in the literature examining the effect of psychological variables on live birth outcomes, the gold standard for ART outcomes [13]. New research using large, representative samples examining the link between psychological variables and lifestyles should be carried out to fully understand the psychological mechanisms that affect infertility and to obtain clinically relevant effect size data.

## Additional files

**Additional file 1: Figure S1.** Prisma flowchart.

**Additional file 2: Table S1.** Study characteristic.

**Additional file 3: Figure S2.** A forest plot of depression data.

**Additional file 4: Figure S3.** A forest plot of state anxiety data.

**Additional file 5: Figure S4.** A forest plot of trait anxiety data.

## Abbreviations

ART: assisted reproductive technologies
IVF: in vitro fertilisation
HFEA: The Human Fertilisation and Embryology Authority
BMI: body mass index
ICSI: intracytoplasmic sperm injection
ZIFT: zygote intrafallopian transfer
