# Supplementary material for: A systematic review and meta-analysis of psychological predictors of successful assisted reproductive technologies
Source: BMC Res Notes. 2017 Dec 7;10:711. doi: 10.1186/s13104-017-3049-z (PMC5719749; doi:10.1186/s13104-017-3049-z)
Supplement: Supplementary file 1 — Additional file 1: Figure S1. Prisma flowchart. [file 13104_2017_3049_MOESM1_ESM.docx]

Studies included in quantitative synthesis (meta-analysis)
(n =22)

Studies included in qualitative synthesis
(n = 22)

Full-text articles assessed for eligibility
(n =169)

Records after duplicates removed
(n = 5189)

Additional records identified through other sources
(n = 27)

Records identified through database searching
(n =5315)

Records excluded
(n =5020)

Full-text articles excluded, with reasons
(N=147)

Do not report depression or state/trait anxiety with ART outcome data=39

Intervention study=36

Not baseline measurement =19

insufficient data reported=14

Other, not relevant, population (e.g., Patients with mental health problems, patients with donated gametes)=13

Not English=10

Multiple reports=5

Not original data=2

Not prospective study=3

Not available =3

Letter=1

Adjusted data=1

Timing of measurement not reported=1

**Figure 1: PRISMA 2009 Flow Diagram of studies included in the psychological meta-analysis**
