# Supplementary material for: A systematic review and meta-analysis of psychological predictors of successful assisted reproductive technologies
Source: BMC Res Notes. 2017 Dec 7;10:711. doi: 10.1186/s13104-017-3049-z (PMC5719749; doi:10.1186/s13104-017-3049-z)
Supplement: Supplementary file 2 — Additional file 2: Table S1. Study characteristic. [file 13104_2017_3049_MOESM2_ESM.docx]

Table1: Study characteristics

| **Authors and country** | **Psychological variable &Measurement** | **Time of assessment and period of enrolment** | **Outcome & assessment of outcome** | **Study Design and sample size** | **Treatment** | **Main findings and author’s conclusion** | **Newcastle-Ottawa Quality Score** |
| --- | --- | --- | --- | --- | --- | --- | --- |
| 1. An et al.2011. China. | *State Anxiety- STAI*  Depression –*BDI* | At initial appointment with fertility clinic nurse  Period of enrolment – 2009-2010 | P  ultrasound scan | Prospective, cohort study  N=264 | IVF,ICSI | **Depression**  Pregnant (n=92, 1.5±1.3)  Not pregnant (n=172, 1.6±1.5).  **State anxiety**  Pregnant (n=92, 36.1±8.8)  Not pregnant (n=172, 37.6±10.0).  No significant differences in depression and state anxiety scores between patents who got pregnant and not pregnant. | Selection ****  Comparability **  Outcome *** |
| 1. Anderheim et al 2005. Sweden. | State Anxiety - *Psychological General Well Being Index (anxiety subscale)*  Depression - *Psychological General Well Being Index (depressed mood subscale)* | At one month before down-regulation.  Period of enrolment – 1999-2002 | P  ultrasound scan | Prospective, cohort study  N=166 | IVF,ICSI | **Depression**  Pregnant (n=58, 15.4±2.2)  Not pregnant (n=81, 15.5±2.5).  **State anxiety**  Pregnant (n=58, 21.0±4.7)  Not pregnant (n=81, 21.8±4.8).  No significant differences in depression and state anxiety scores between patents who got pregnant and not pregnant | Selection ****  Comparability **  Outcome *** |
| 1. Boivin & Takefman 1995. Canada. | State Anxiety - *STAI*  Trait Anxiety – *STAI* | At patient screening before IVF  Period of enrolment – 1.5 years study period. | P  HCG test | Prospective, cohort study  N=40 | IVF | **State anxiety**  Positive test (n=17, 34.09±8.2)  Negative test (n=23, 37.92±12.4).  **Trait anxiety**  Positive test (n=17, 35.02±9.7)  Negative test (n=23, 36.91±10.0).  No significant differences in state and trait anxiety scores between patents who got pregnant and not pregnant. | Selection ***  Comparability **  Outcome ** |
| 1. de Klert et al 2008. The Netherlands. | State Anxiety – *HADS*  Depression –*HADS* | IVF consultation 6 weeks before treatment.  Period of enrolment – 2002-2004 | LB  >=37 weeks gestation | Prospective, cohort study  N=289 | IVF,ICSI | **Depression**  LB (n=73, 2.5±2.8)  No LB (n=216, 2.6±2.6).  **State anxiety**  LB (n=73, 5.1±3.9)  No LB (n=216, 5.0±3.4).  No significant differences in depression and state anxiety scores between patents who achieved LB and patients who did not. | Selection ***  Comparability  Outcome *** |
| 1. Demyttenaere et al 1992. Belgium. | Depression - *Zung Depression scale* | First visit to clinic during IVF cycle (day 4-5)  Period of enrolment not reported. | P  HCG test | Prospective, cohort study  N=40 | IVF | **Depression**  Pregnant (n=10, 42.0±2.7)  Not pregnant (n=30, 47.4±1.7).  Although depression scores for pregnant and not pregnant groups were not significantly different, however, depression and other coping behaviours were significant predictors which accounted for 27% variance in pregnancy chances. | Selection ***  Comparability **  Outcome ** |
| 1. Demyttenaere et al 1998. Belgium. | Depression - *Zung Depression scale* | First visit to clinic during IVF cycle (day 3).  Period of enrolment not reported. | P  Ultrasound scan | Prospective, cohort study  N=98 | IVF | **Depression**  Pregnant (n=23, 52.8±8.8)  Not pregnant (n=75, 52.4±10.1).  Depression scores for pregnant and not pregnant groups were not significantly different. | Selection ****  Comparability *  Outcome *** |
| 1. Ebbesen et al 2009. Denmark. | Depression – *BDI* | Before follicle scan.  Period of enrolment – 2001-2006 | P  Ultrasound scan | Prospective, cohort study  N=809 | IVF | **Depression**  Pregnant (n=217, 7.2±6.1)  Not pregnant (n=592, 7.2±6.5).  Depression scores for pregnant and not pregnant groups were not significantly different. | Selection ****  Comparability  Outcome *** |
| 1. Gurhan et al 2009. Turkey. | Depression –*BDI*  State Anxiety - *STAI*  Trait Anxiety – *STAI* | At initial appointment with fertility clinic nurse  Period of enrolment -September 2004 and July 2005 | P  HCG test | Prospective, cohort study  N=80 | IVF | **Depression**  Positive test (n=39, 10.1±5.2)  Negative test (n=41, 12.7±6.2).  **State anxiety**  Positive test (n=39, 45.0±4.6)  Negative test (n=41, 47.6±7.8).  **Trait anxiety**  Positive test (n=39, 38.3±4.6)  Negative test (n=41, 40.6±8.1).  Only depression at baseline was associated with negative pregnancy outcomes. | Selection ****  Comparability  Outcome ** |
| 1. Lancastle & Boivin 2005. UK. | State Anxiety - *STAI*  Trait Anxiety – *STAI* | 3 months before treatment.  Period of enrolment not reported. | P  HCG test | Prospective, cohort study  N=76 | IVF | **State anxiety**  Positive test (n=13, 36.0±12.3)  Negative test (n=63, 41.0±11.2).  **Trait anxiety**  Positive test (n=13, 37.2±9.46)  Negative test (n=63, 39.19±9.96). | Selection ***  Comparability  Outcome ** |
| 1. Li et al 2011. China | State Anxiety –*Zung self rating anxiety scale (SAS)*  Depression –*Zung self rating depression scale (SDS)* | First day of down-regulation.  Period of enrolment 2007-2008. | P  Ultrasound scan | Prospective, cohort study  N=107 | IVF | **Depression**  Pregnant (n=50, 52.66±12.34)  Not pregnant (n=57, 54.06±11.34).  **State anxiety**  Pregnant (n=50, 39.66±8.04)  Not pregnant (n=57, 39.30±7.93).  Depression and anxiety scores for pregnant and not pregnant groups were not significantly different. | Selection ****  Comparability **  Outcome *** |
| 1. Lintsen et al 2009. The Netherlands. | State Anxiety - *STAI*  Depression –*BDI* | 1-2 months before treatment.  Period of enrolment 2002-2004. | P  Ultrasound scan | Prospective, cohort study  N=690 | IVF,ICSI | **Depression**  Pregnant (n=196, 1.2±1.8)  Not pregnant (n=494, 1.4±2.4).  **State anxiety**  Pregnant (n=196, 176±4.7)  Not pregnant (n=494, 17.7±5.0).  Depression and anxiety scores for pregnant and not pregnant groups were not significantly different. | Selection ****  Comparability **  Outcome ** |
| 1. Merari et al 2002. Israel. | State Anxiety - *STAI*  Trait Anxiety - *STAI*  Depression - *Lubin’s Depression adjective checklist scale (DACL)* | 10-15 days before initiation of treatment.  Period of enrolment not reported. | P  Not specified | Prospective, cohort study  N=113 | IVF | **Depression**  Pregnant (n=23, 11.35±5.57)  Not pregnant (n=90, 9.87±4.19).  **State anxiety**  Pregnant (n=23, 43.04±15.5)  Not pregnant (n=90, 39.18±10.6).  **Trait anxiety**  Pregnant (n=23, 41.04±10.9)  Not pregnant (n=90, 41.44±8.11).  Depression and anxiety scores for pregnant and not pregnant groups were not significantly different. | Selection ***  Comparability *  Outcome ** |
| 1. Pasch et al 2012. US. | Depression- *Centre for Epidemiological Studies Depression Scale (CES-D)*  *State Anxiety – STAI* | Before the start of IVF cycle.  Period of enrolment 2000-2004. | P & LB  Delivery or ultrasound scan | Prospective, cohort study  N=202 | IVF | **Depression**  Pregnant (n=57, 11.29±8.77)  Not pregnant (n=145, 12.39±10.23).  **State anxiety**  Pregnant (n=57, 39.96±11.67)  Not pregnant (n=145, 41.41±11.55).  Depression and anxiety scores for pregnant and not pregnant groups were not significantly different. | Selection ***  Comparability  Outcome ** |
| 1. Sanders & Bruce, 1999. Australia. | State Anxiety - *STAI*  Trait Anxiety – *STAI* | 1-3 months before treatment.  Period of enrolment 1990-1993. | P  Ultrasound scan | Prospective, cohort study  N=90 | IVF/GIFT/FET | **State anxiety**  Pregnant (n=32, 34.78±10.17)  Not pregnant (n=58, 38.97±10.36).  **Trait anxiety**  Pregnant (n=32, 37.1±10.34)  Not pregnant (n=58, 38.38±7.93). | Selection ***  Comparability  Outcome *** |
| 1. Slade et al 1997. UK. | Depression –*BDI* | Day 1 of first cycle.  Period of enrolment – 33 months study period. | P  Not specified | Prospective, cohort study  N=144 | IVF | **Depression**  Pregnant (n=42, Not depressed = 67%, mildly depressed = 26% and moderately depressed = 7%).  Not pregnant (n=102, Not depressed = 72%, mildly depressed = 21% and moderately depressed = 7%).  Rates of depression for pregnant and not pregnant groups were not significantly different. | Selection ****  Comparability  Outcome * |
| 1. Sohrabvand et al 2009. Iran. | State Anxiety - *Iranian Cattle Anxiety scale**  Depression –*BDI* | Before down-regulation.  Period of enrolment 2006-2007. | P  Not specified | Prospective, cohort study  N=106 | ICSI | **Depression**  Pregnant (n=25, Not depressed = 96%, mildly depressed = 4%)  Not pregnant (n=81, Not depressed = 61.7%, mildly depressed = 32.4% and moderately depressed = 4.9%, severely depressed = 1.2%).  **State Anxiety**  Pregnant (n=25, Not anxious =84%, mildly anxious = 8% and moderately anxious = 8%).  Not pregnant (n=81, Not anxious = 8.6%, mildly anxious = 30.9% and moderately anxious = 40.7%, severely anxious = 19.7%).  Rates of depression and state anxiety for pregnant and not pregnant groups were significantly different. | Selection ***  Comparability *  Outcome ** |
| 1. Terzioglu et al 2016. Turkey. | Depression –*BDI*  State Anxiety - *STAI*  Trait Anxiety – *STAI* | Before the start of IVF cycle  Data collection – January-December 2009 | P  HCG test | Prospective, cohort study  N=217 | ICSI | **Depression**  Positive test (n=70, 14.06±9.52)  Negative test (n=147, 17.45±10.68).  **State anxiety**  Positive test (n=70, 41.97±5.55)  Negative test (n=147, 41.95±5.39).  **Trait anxiety**  Positive test (n=70, 48.84±5.50)  Negative test (n=147, 49.97±6.63).  Only depression at baseline was associated with negative pregnancy outcomes. | Selection ***  Comparability  Outcome ** |
| 1. Thiering et al 1993. Australia. | Depression - *Centre for Epidemiological Studies Depression Scale (CES-D)* | One month before treatment.  Period of enrolment -12 months study. | P  Ultrasound scan | Prospective, cohort study  N=97 (first time patients only) | IVF | **Depression**  Pregnant n=31 (not depressed n=27 and depressed n =4)  Not pregnant n=66 (not depressed n=56 and depressed n=10).  Rates of depression for pregnant and not pregnant groups were not significantly different. | Selection ****  Comparability  Outcome *** |
| 1. Turner et al 2013. US. | State Anxiety - *STAI*  Trait Anxiety – *STAI* | Period of enrolment June 2009-September 2009. | P  ultrasound scan | Prospective, cohort study.  N=36 | IVF | **State anxiety**  Pregnant (37.53±12.33)  Not pregnant (43.57±14.44).  **Trait anxiety**  Pregnant (35.93±11.00)  Not pregnant (38.86±10.88).  No significant differences in state and trait scores between patents who got pregnant and not pregnant. | Selection ***  Comparability  Outcome ** |
| 1. Verhaak et al. 2001. The Netherlands. | State Anxiety - *STAI*  Depression – *BDI* | 3-10 days before first cycle.  Period of enrolment not reported. | P  Ultrasound scan | Prospective, cohort study  N=206 | IVF,ICSI | **Depression**  Pregnant (n=59, 4.1±3.6)  Not pregnant (n=148, 6.4±5.8).  **State anxiety**  Pregnant (n=59, 35.6±8.3)  Not pregnant (n=148, 38.0±10.9).  There were significant differences in depression scores between women who got pregnant and women who did not. There was a trend towards differences in state anxiety score. | Selection ****  Comparability **  Outcome ** |
| 1. Visser et al 1994. The Netherlands. | State Anxiety - *STAI*  Depression - *Hopkins symptom checklist* | Before treatment but not specified when.  Period of enrolment 1986-1989. | P  Not specified | Prospective, cohort study  N=65 | IVF | **Depression**  Pregnant (n=12, 25.0±12.4)  Not pregnant (n=53, 22.4±9.2).  **State anxiety**  Pregnant (n=12, 43.6±13.6)  Not pregnant (n=53, 43.9±11.4). | Selection ****  Comparability  Outcome * |
| 1. Yilmaz et al 2015. Turkey. | Depression – *BDI* | First visit to IVF clinic.  Period of enrolment January 2013-August 2013. | P  ultrasound scan | Prospective, cohort study.  N=83 | IVF | **Pregnant** (n=26) **BD**I scores <16 = 19 (73.1%) and BDI scores >16 = 7 (26.9%).  **Not pregnant** (n=57) **BDI** scores <16 = 29 (50.9%) and BDI scores >16 = 28 (49.1%).  No significant differences in clinical depression rates between patents who got pregnant and not pregnant. | Selection ***  Comparability *  Outcome *** |

**Note ICSI = intracytoplasmic sperm injection; IVT = in vitro fertilisation; LB = live birth outcome data; P = pregnancy outcome data. The sample size refers to data that is extracted from the papers and used in the meta-analysis.**
