# Supplementary figures and images for: A systematic review and meta-analysis of psychological predictors of successful assisted reproductive technologies
Source: BMC Res Notes. 2017 Dec 7;10:711. doi: 10.1186/s13104-017-3049-z (PMC5719749; doi:10.1186/s13104-017-3049-z)

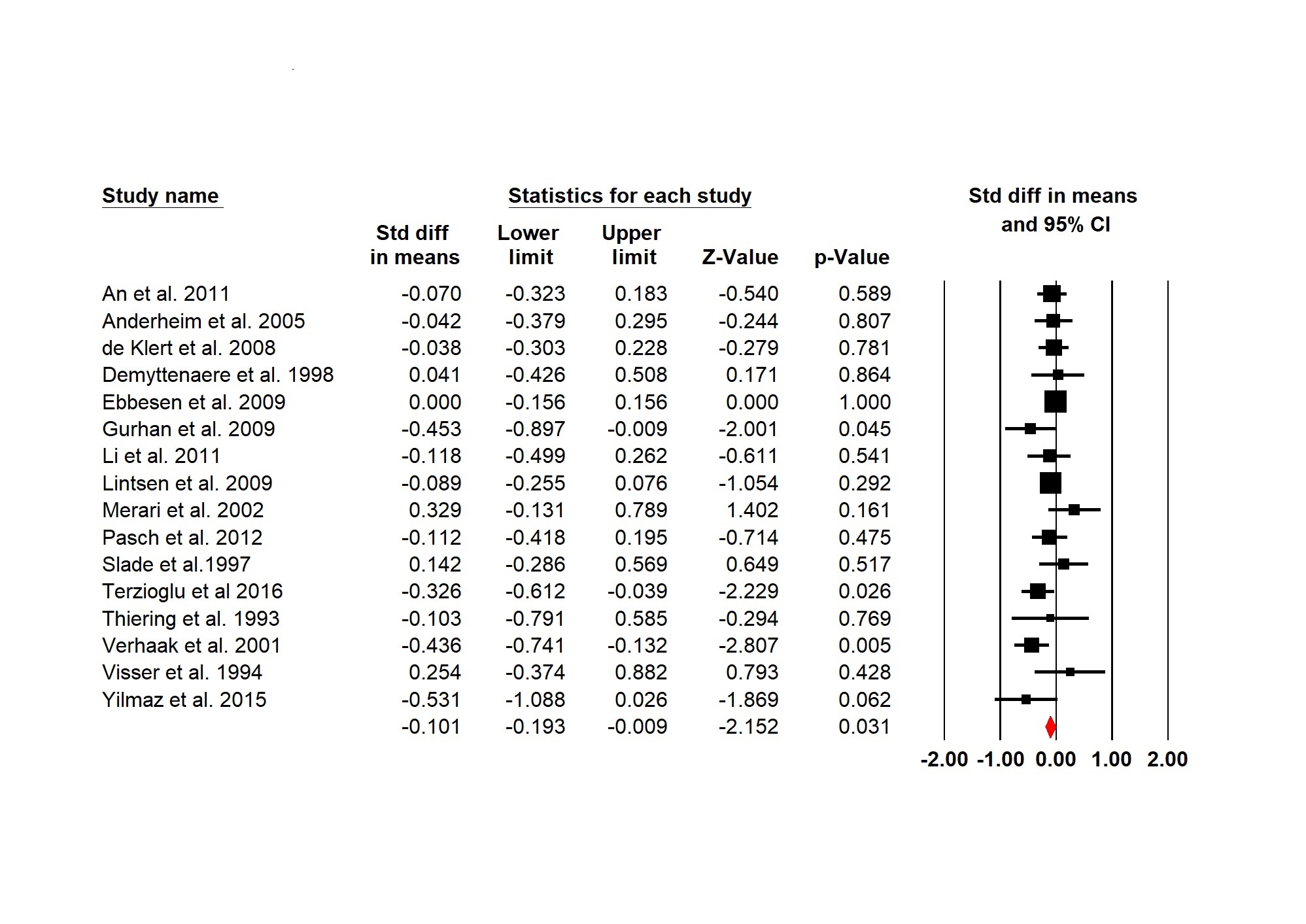

Supplement: Supplementary file 3 — Additional file 3: Figure S2. A forest plot of depression data. [file 13104_2017_3049_MOESM3_ESM.jpg]

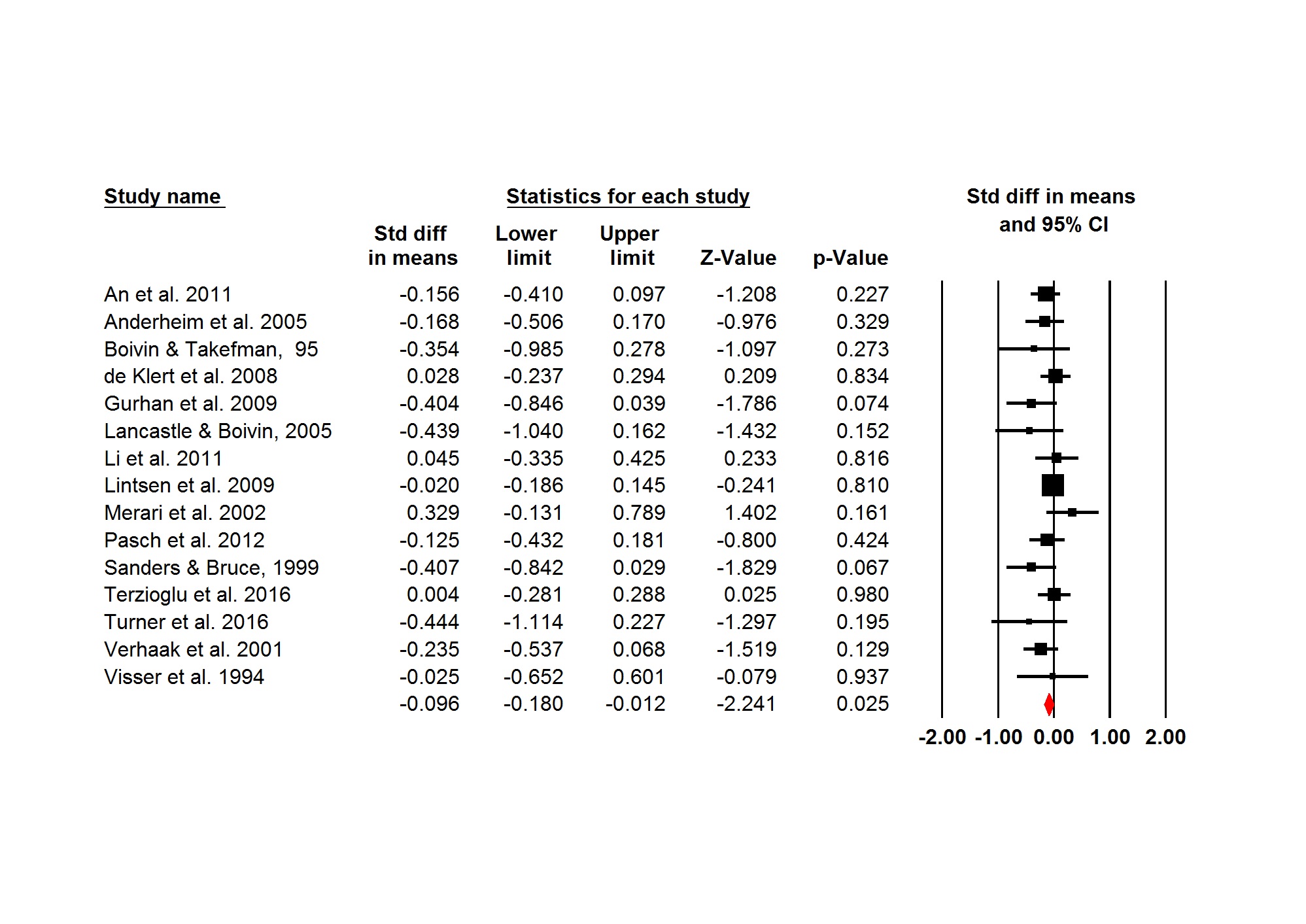

Supplement: Supplementary file 4 — Additional file 4: Figure S3. A forest plot of state anxiety data. [file 13104_2017_3049_MOESM4_ESM.jpg]

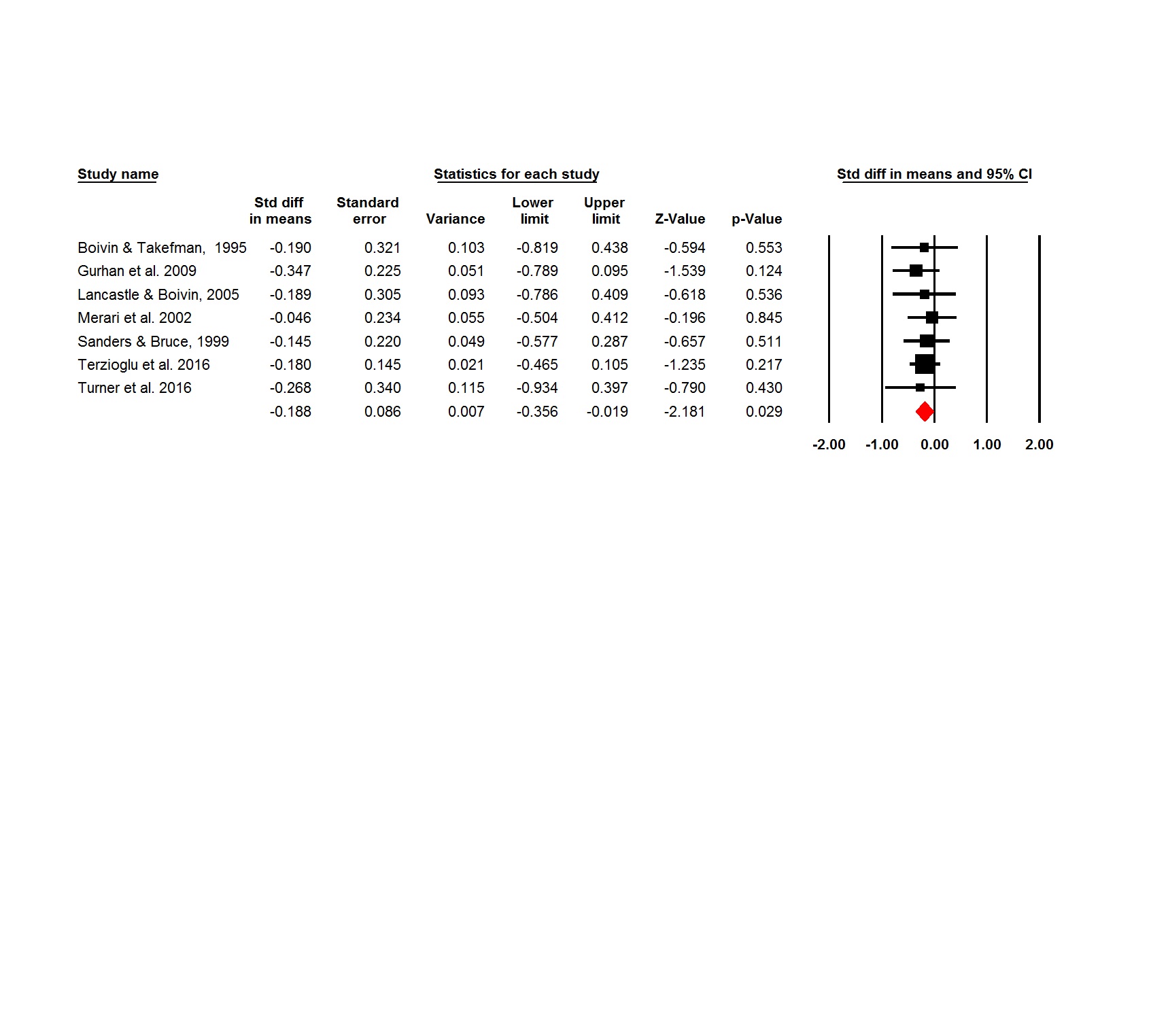

Supplement: Supplementary file 5 — Additional file 5: Figure S4. A forest plot of trait anxiety data. [file 13104_2017_3049_MOESM5_ESM.jpg]
